# Metagenomes and Assembled Genomes from Diarrhea-Affected Cattle (Bos taurus)

**DOI:** 10.1128/MRA.01411-20

**Published:** 2021-02-18

**Authors:** Tshepiso Pleasure Ateba, Kazeem Adekunle Alayande, Mulunda Mwanza

**Affiliations:** a Department of Animal Health, Faculty of Natural and Agricultural Sciences, Mafikeng Campus, North West University, Mmabatho, Mafikeng, South Africa; b Department of Microbiology, Faculty of Natural and Agricultural Sciences, Mafikeng Campus, North West University, Mmabatho, Mafikeng, South Africa; Indiana University, Bloomington

## Abstract

The *de novo* metagenome assembly for C1-TPA is 68,577,389 bp long spread over 10,108 contigs, while that of C3-TPA is 55,517,929 bp distributed over 9,415 contigs. A total of 8 metagenome-assembled genomes (MAGs) were extracted from C1-TPA, and 10 were extracted from C3-TPA. Both samples have a *Flavobacterium* sp. and a *Pseudomonas* sp. in common among their bacterial communities.

## ANNOUNCEMENT

Diarrheal disease remains a major cause of morbidity and mortality in the developing world; cattle and young calves are highly susceptible to enteric infections caused by various pathogens (1). Diarrheal samples, C1-TPA and C3-TPA, were collected from affected cattle (Bos taurus) directly from the rectum with sterile nitrile gloves, at Lokaleng Village in Mafikeng, South Africa (25.82°S, 25.58°E). About 150 mg of each of the fecal samples was apportioned for metagenome DNA extraction using a Quick-DNA fecal/soil microbe miniprep kit (Zymo Research Corp., USA). The library was prepared with a Nextera DNA Flex library preparation kit (Illumina) using Nextera DNA CD index adapters (96 indexes plated). The final concentrations of the libraries (70.80 ng/μl for both C1-TPA and C3-TPA) were measured using the Qubit double-stranded DNA (dsDNA) high-sensitivity (HS) assay kit (Life Technologies), and the average library sizes (521 bp and 523 bp for C1-TPA and C3-TPA, respectively) were determined using the Agilent 2100 bioanalyzer. The libraries were then pooled in equimolar ratios of 8.0 pM and sequenced on an Illumina NovaSeq 6000 system. The numbers of reads generated thereafter were 14,302,284 and 14,431,130 for samples C1-TPA and C3-TPA, respectively. The read length used in the library preparation was 2 × 150 bp, and the coverage of the sequence was 29× for C1-TPA and 36× for C3-TPA.

The sequenced data were assessed and filtered with Trimmomatic v0.36 (2) for low-quality reads and adapter fragments. The adapter sequences were clipped using a mismatch value of 2, a palindrome clip threshold of 30, and a simple clip threshold of 10. The taxonomy of the metagenomes was determined using Kaiju v1.7.2 (3) and GOTTCHA2 v2.1.6. The *de novo* metagenome assembly was constructed with metaSPAdes v3.13.0 (4). Each of the metagenome assemblies was binned using MaxBin 2 v2.2.4 (5) and CONCOCT v1.1 set at different modes—Bowtie2-default and Bowtie2-verysensitive, respectively—and BBMap. The binned contigs were then optimized to exclude bins that have low completeness and high contamination using DAS Tool v1.2. Each bin was then extracted as a metagenome-assembled genome (MAG) and assessed for quality control using CheckM v1.0.18 (6). The taxonomic assignments were obtained for the MAGs based on the genome taxonomy database using GTDB-Tk v1.1.0 (7) and Microbial Genomes Atlas (MiGA) (8); where the taxonomic assignment differs, identity with a higher average nucleotide identity (ANI) percentage was selected.

The MAGs were annotated using the NCBI Prokaryotic Genome Annotation Pipeline (PGAP) v4.12 (9). The acquired drug-resistant genes were determined using ResFinder v4.0 (10). Most of the software was accessed through the KBase workspace service v0.11.1 (11), except for MiGA, PGAP, and ResFinder. Default parameters were used for all software engaged in the analysis except where stated otherwise.

The *de novo* metagenome assembly for C1-TPA has 68,577,389 bp distributed over 10,108 contigs, and the metagenome assembly was binned into eight MAGs. For sample C3-TPA, the assembly size is 55,517,929 bp distributed across 9,415 contigs. Ten different MAGs were extracted from the metagenome. Not all the contigs were binned into the MAGs in both samples. Both samples have *Flavobacterium* spp. (*Flavobacterium* sp. strain N1CT and *Flavobacterium* sp. strain NTP45) and *Pseudomonas* spp. (*Pseudomonas* sp. strain N17CT and Pseudomonas stutzeri NTP17) in common (Table 1).

**TABLE 1:** Characteristics and accession numbers of the metagenome-assembled genomes^*a*^

Physical sample	MAG identity	Genome size (bp)	Total CDS	No. of contigs	*N*_50_ (bp)	G+C content (%)	CMP (%)	CNT (%)	Genome accession no.
C1-TPA	*Flavobacterium* sp. isolate N1CT	2,649,527	2,357	49	269,215	42.12	99.65	0.00	JADGMX000000000
	*Cellulomonas* sp. isolate N5CT	4,210,595	3,806	38	144,832	74.00	99.21	1.73	JADGMY000000000
	*Brevundimonas* sp. isolate N6CT	2,167,354	2,426	375	6,091	70.32	76.06	1.14	JADGMZ000000000
	*Salinibacterium* sp. isolate N14CT	2,804,916	2,675	23	385,244	68.00	98.55	1.52	JADGNA000000000
	*Pseudomonas* sp. isolate N17CT	4,778,161	5,202	1151	4,176	62.97	81.61	28.55	JADGNB000000000
	*Rhodococcus* sp. isolate N19CT	4,327,264	4,079	119	35,912	67.36	97.11	0.88	JADGNC000000000
	*Pseudomonas* sp. isolate N24CT	4,040,679	3,727	45	149,313	61.02	99.39	3.93	JADGND000000000
	*Devosia* sp. isolate N26CT	3,978,489	3,820	74	140,542	62.26	98.69	0.00	JADGNE000000000
C3-TPA	*Planococcus* sp. (in: Bacteria) isolate NTP4	2,417,639	2,663	476	4,504	45.23	62.20	1.88	JADMKI000000000
	*Proteiniphilum* sp. isolate NTP5	3,297,167	2,664	237	20,538	47.25	98.27	0.70	JADMKJ000000000
	*Comamonas* sp. isolate NTP6	3,198,324	2,949	61	75,187	57.54	97.33	0.46	JADMKK000000000
	*Candidimonas* sp. isolate NTP16	3,757,657	3,549	88	79,153	59.04	99.59	0.83	JADMKL000000000
	Pseudomonas stutzeri NTP17	3,293,093	3,121	159	26,172	60.95	95.98	1.23	JADMKM000000000
	*Patulibacter* sp. isolate NTP18	3,375,244	3,322	297	14,726	71.72	93.16	1.79	JADMKN000000000
	*Chryseobacterium* sp. isolate NTP27	2,041,705	2,007	187	11,889	39.88	94.00	1.96	JADMKO000000000
	*Fermentimonas* sp. isolate NTP30	2,897,645	2,309	13	651,827	36.07	100.0	1.64	JADMKP000000000
	*Sphingorhabdus* sp. isolate NTP38	2,673,275	2,644	243	12,850	54.27	93.14	1.91	JADMKQ000000000
	*Flavobacterium* sp. isolate NTP45	2,497,823	2,452	275	9,398	43.55	92.90	1.00	JADMKR000000000

a CMP, completeness; CNT, contamination; CDS, coding sequence. The values for completeness and contamination of each MAG were determined using CheckM v1.0.18, while the genome sizes were determined using v1-KBaseGenomeAnnotations.Assembly-5.0 and the NCBI Prokaryotic Genome Annotation Pipeline (PGAP).

Ethical clearance for the study was approved by the Research Ethics Committee of North West University, South Africa (NWU-00160-14-A9).

### Data availability.

All data were deposited under the GenBank BioProject number PRJNA661076. The whole-genome shotgun projects have been deposited in DDBJ/ENA/GenBank under the accession numbers JADGMW000000000 and JADKLW000000000. The versions described in this paper are the first versions, JADGMW000000000.1 and JADKLW000000000.1. The SRA accession numbers for the raw reads are SRX9212776 and SRX9218438 for samples C1-TPA and C3-TPA, respectively.
